# Modulating Antimicrobial Activity and Structure of the Peptide Esc(1‐21) via Site‐Specific Isopeptide Bond Formation

**DOI:** 10.1002/psc.70048

**Published:** 2025-08-06

**Authors:** Bruno Casciaro, Daniel Ben Hur, Daniela Roversi, Carlo Vetrano, Edo Kiper, Giacomo Cappella, Federico Carneri, Eeva Tortellini, Lorenzo Stella, Neta Regev‐Rudzki, Yechiel Shai, Maria Luisa Mangoni

**Affiliations:** ^1^ Laboratory Affiliated to Pasteur Italia‐Fondazione Cenci Bolognetti, Department of Biochemical Sciences Sapienza University of Rome Rome Italy; ^2^ Department of Biomolecular Sciences, Faculty of Biochemistry Weizmann Institute of Science Rehovot Israel; ^3^ Department of Chemical Science and Technologies University of Rome Tor Vergata Rome Italy; ^4^ Photoinduced Processes and Technologies Doctoral School, Department of Chemistry, Biology and Biotechnology Perugia University Perugia Italy

## Abstract

Antimicrobial peptides (AMPs) represent valid alternatives to conventional antibiotics primarily due to their mechanism of action, which consists of cytoplasmic membrane disruption. However, their clinical application is often limited by cytotoxicity at high concentrations and low intrinsic biostability. To address these limitations, various biochemical approaches have been explored. In recent years, the frog‐skin derived AMP Esc(1‐21) has been extensively characterized for its potent antimicrobial activity, especially against Gram‐negative bacteria, both in vitro and in vivo. In this study, we designed and synthesized novel Esc(1‐21) analogs in which a single isopeptide bond was introduced in place of a conventional peptide bond at specific positions within the sequence. The resulting five analogs were evaluated for their (i) chemical and structural properties, (ii) resistance to proteolytic degradation, (iii) antimicrobial and antibiofilm activities, (iv) hemolytic and cytotoxic effects, and (v) ability to perturb bacterial cytoplasmic membranes. Among these, Esc(1‐21)ε20 showed the most promising features, maintaining antimicrobial and antibiofilm activities comparable to those of the parent peptide while exhibiting lower cytotoxicity towards eukaryotic cells at higher concentrations and greater resistance to enzymatic degradation. These findings highlight Esc(1‐21)ε20 as an attractive lead candidate for the development of new antibiotic therapeutics.

## Graphical abstract

The introduction of an isopeptide bond at the lysine residues in the backbone of Esc(1‐21) led to the generation of five analogs. Among these, Esc(1‐21)ε20 showed the most promising features, having antimicrobial activity comparable with those of the parent peptide, a lower cytotoxicity, and a higher stability to proteolytic degradation.

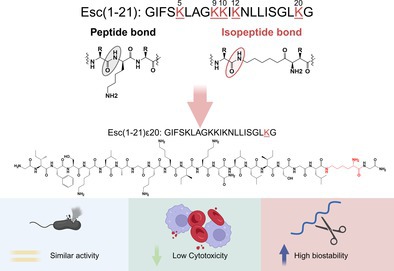

## 1 Introduction

Bioactive peptides are a class of compounds consisting of short amino acid sequences, capable of performing various physiological functions, including the regulation of blood pressure, immune activity, glucose metabolism, protection against oxidative stress, and, last but not least, antimicrobial activity [1]. Among these bioactive compounds, antimicrobial peptides (AMPs) hold great promise in the current post‐antibiotic era, during which only a small number of new antibiotics are being introduced to the market [2]. AMPs are gene‐encoded cationic molecules, typically composed of about 10–50 amino acids and with various amphipathic secondary structures [3]. Their cationicity and amphipathicity are considered key physicochemical properties that make AMPs active against biological membranes [4], particularly those of bacteria, which are rich in anionic phospholipids [5]. Through a mechanism involving membrane destruction by pore formation or micellization, AMPs represent a valid alternative to conventional antibiotics, which generally act via specific molecular targets prone to mutation [6, 7, 8]. In recent years, a frog‐skin derived AMP, named Esculentin‐1a(1‐21)NH_2_ [Esc(1‐21)], has been characterized for its potent antimicrobial activity, especially against Gram‐negative bacteria, both in vitro and in vivo [9, 10, 11]. Furthermore, several chemical modification strategies have been adopted to broaden the spectrum of action of the peptide (e.g., against Gram‐positive bacteria), to increase its resistance to proteolytic degradation, and to reduce its cytotoxicity at high concentrations. These include the replacement of two l‐residues with their corresponding d‐enantiomers [12]; the replacement of certain residues with the nonproteinogenic α‐aminoisobutyric acid (Aib) at positions 1, 10, and 18 [13], as well as the strategic position 8 [14]. In this work, we adopted a different approach by synthesizing Esc(1‐21) analogs in which the typical peptide bond between two residues was replaced with an isopeptide bond. Isopeptide bond formation involves lysine residues forming covalent linkages via their ε‐amino groups. Generally, this noncanonical bond enhances resistance to proteolytic degradation, stabilizes tertiary and quaternary structures, and facilitates the covalent attachment of proteins to biological surfaces [15, 16]. Naturally occurring isopeptide bonds have been identified in systems such as the HK97 bacteriophage capsid, Gram‐positive bacterial pili, and the ubiquitin‐proteasome pathway in eukaryotic cells [17, 18, 19]. In these contexts, isopeptide bonds are typically formed between separate protein domains or subunits. In contrast, our work explores the direct incorporation of this noncanonical bond within the backbone of the AMP sequence, representing an innovative application of isopeptide bond chemistry in peptide engineering. In fact, to the best of our knowledge, very few studies have explored isopeptide bond incorporation in AMPs [20, 21, 22, 23], confirming the intriguing strategy of this biochemical approach. Considering the presence of five lysine residues in the primary structure of Esc(1‐21), five peptides with site‐specific isopeptide bond switches have been synthesized and characterized for their (i) chemical properties, (ii) structural properties, (iii) stability to proteolytic degradation, (iv) antimicrobial and antibiofilm activity, (v) hemolytic and cytotoxic effect, and (vi) ability to perturb bacterial cytoplasmic membranes.

## 2 Materials and Methods

### 2.1 Materials

9‐Fluorenylmethoxylcarbonyl (Fmoc)‐rink amide methyl‐benzhydryl amine (MBHA) resin, ethyl cyanohydroxyiminoacetate (Oxyma) and Fmoc amino acids were purchased from Calibochem/Novabiochem AG (Laufelfinger, Switzerland). Acetonitrile (ACN), methanol, *N*,*N*‐dimethylformamide (DMF), dichloromethane (DCM), diethyl ether (Et_2_O), piperidine, and trifluoroacetic acid (TFA) were provided by Bio‐Lab Ltd, (Israel). *N*,*N*′‐Diisopropylcarbodiimide (DIC) and triethylsilane (TES) were purchased from Sigma‐Aldrich (Israel). Sodium‐dodecylsulfate (SDS) was obtained from Cambridge Isotope Laboratories (Tewksbury, MA, USA); 3‐(4,5‐dimethylthiazol‐2‐yl)‐2,5‐diphenyltetrazolium bromide (MTT) was supplied by Merck (Darmstadt, Germany) and Cell Counting Kit 8 (CCK‐8) was purchased from MedChemExpress (Monmouth Junction, NJ, USA).

### 2.2 Peptide Synthesis

Peptides were synthesized using an automated microwave‐assisted solid‐phase peptide synthesizer (Liberty Blue, CEM Corporation) on 0.57 mmol/g MBHA resin, using the Fmoc solid‐phase strategy [24, 25]. Isopeptide bond formation was achieved by incorporating Boc‐L‐Lys(Fmoc)‐OH, enabling coupling through the ε‐amino group of lysine, in contrast to the conventional Fmoc‐L‐Lys(Boc)‐OH used for α‐peptide bond formation. The resin‐bound peptide was washed thoroughly with DMF, then with DCM, and dried under nitrogen flow. Cleavage of the peptide from the resin was done by adding 95% TFA, 2.5% ddH_2_O, and 2.5% TES. The resin was filtered from the mixture, and the peptide was precipitated from the mixture using cold Et_2_O.

### 2.3 Peptide Purification and Characterization

The purification of the peptides was performed by reverse‐phase high‐performance liquid chromatography (RP‐HPLC) on an Agilent Technologies 1260 Infinity II spectrometer with a reversed‐phase Vydac C_4_ column at a flow rate of 1.8 mL/min and monitored with an ultraviolet (UV) detector at 215 nm. Linear gradients of 10%–90% ACN in ddH_2_O containing 0.1% (v/v) TFA were used for peptide purification for 40 min. Final products were obtained by freeze‐drying the collected pure fractions. The purity of the peptides was validated with a C18 reversed‐phase column (Thermo Fisher Scientific, 250 mm × 4.6 mm, 5 μm particle size) at a flow rate of 0.6 mL/min using a gradient of 10%–90% ACN in ddH_2_O containing 0.1% (v/v) TFA for 40 min with UV detection at 215 nm. Incorporation of the isopeptide bond was verified as done previously [22], based on differences in hydrophobicity compared between the peptide parental and analogs, resulting in distinct retention times under identical conditions. Molecular weights were determined by time‐of‐flight mass spectrometry (TOF‐MS), confirming the expected mass and structural integrity of each peptide. The purity of all peptides examined was > 95%.

### 2.4 Microbial and Eukaryotic Cells

The following strains from American Type Culture Collection (ATCC) were used in this study: the Gram‐negatives *Escherichia coli* ATCC 25922, *Pseudomonas aeruginosa* ATCC 27853, *Pseudomonas aeruginosa* ATCC 15692; and the Gram‐positives *Staphylococcus epidermidis* ATCC 12228 and *Staphylococcus aureus* ATCC 25923. For the cytotoxicity assay, HaCaT cells (AddexBio San Diego, CA, United States) were used: the cells were grown in Dulbecco's modified Eagle's medium supplemented with 4 mM glutamine (DMEMg), 10% heat‐inactivated fetal bovine serum (FBS), and 0.1 mg/mL penicillin and streptomycin. The cell culture was maintained in a humidified incubator at 37°C and 5% CO_2_.

### 2.5 Antimicrobial and Antibiofilm Activity

The antimicrobial activity of Esc(1‐21) analogs was assessed by determining the minimal inhibitory concentration (MIC) against a panel of representative Gram‐negative and Gram‐positive bacteria. Bacteria were grown at 37°C until an optical density (OD) of 0.8 was reached at 590 nm. The bacteria were then diluted in Mueller‐Hinton (MH) broth at a concentration of 2 × 10^6^ colony‐forming units (CFU)/mL. Aliquots (50 μL) were added to 50 μL of MH containing serial two‐fold dilutions of the peptides, which had been previously prepared in the wells of a 96‐well plate, achieving a final cell concentration of 10^6^ CFU/mL. The plate was incubated at 37°C for 16–18 h and the MIC was defined as the lowest concentration capable of visually inhibiting microbial growth. To evaluate the antibiofilm activity of the tested peptides, bacteria were diluted to a cell density of 10^6^ CFU/mL and aliquots of 100 μL were dispensed into the wells of a 96‐multiwell plate and incubated for 20 h at 37°C to allow biofilm formation. Planktonic cells were then removed, and each well was washed twice with 150 μL of phosphate buffered saline (PBS). Each well was filled with PBS supplemented with different 2‐fold serial dilutions of the tested peptides and the plate was incubated for 2 h at 37°C. After two washes with PBS, biofilm viability was assessed by adding 150 μL of MTT (0.5 mg/mL). The plate was incubated for 4 h at 37°C and the reaction was then stopped by adding SDS (final concentration equal to 5% v/v). The absorbance of each well was measured at 570 nm with a microplate reader (Infinite M200; Tecan, Salzburg, Austria), and the percentage of biofilm viability was calculated with respect to the untreated samples.

### 2.6 Membrane Permeabilization Assay

To investigate the potential of Esc(1‐21) analogs to permeabilize cytoplasmic membranes of Gram‐positive and Gram‐negative bacteria, a Sytox Green assay was performed using *E. coli* ATCC 25922 and *S. epidermidis* ATCC 12228 as representative strains. Bacterial suspensions were prepared at approximately 10^7^ CFU/mL in PBS and incubated with 1 μM Sytox Green for 5 min in the dark. After that, peptides were added at concentrations ranging from 0.78 to 50 μM (time = 0). Fluorescence intensity (λ_exc_ = 485 nm, λ_ems_ = 535 nm), which reflects the binding of the dye to intracellular DNA after membrane permeabilization, was monitored over 30 min using a microplate reader (Infinite M200, Tecan, Salzburg, Austria) at 37°C. Experiments were performed in triplicate. Bacterial cells treated with vehicle (water) were used as a control.

### 2.7 Hemolytic Assay

The hemolytic activity of Esc(1‐21) analogs was assessed by quantifying the release of hemoglobin resulting from the disruption of defibrinated sheep red blood cells (RBCs) (OXOID, SR0051D, Milan, Italy). RBCs were washed twice in 0.9% (w/v) NaCl. Aliquots of red blood cell suspension were adjusted to an OD of 0.5 at 500 nm in 0.9% (w/v) NaCl and incubated with peptide solutions at concentrations ranging from 0.78 to 50 μM for 30 min at 37°C with gentle agitation. After centrifugation, the samples were centrifuged at 900 × *g* for 5 min, and hemoglobin released in the supernatant was quantified by measuring absorbance at 415 nm using a microplate reader (Infinite M200; Tecan, Salzburg, Austria) and compared with the complete lysis of erythrocytes (100%) in ddH_2_O. Cells treated with vehicle (ddH_2_O) served as the negative control. The data were reported as the mean ± the standard error of the mean (SEM) of three independent experiments.

### 2.8 Cell Viability Assay

The viability of HaCaT cells was assessed using the CCK‐8 assay as previously reported [9, 26]. Briefly, 100 μL of cell suspensions (10^4^ cells) were added to each well of a 96‐well plate and incubated for approximately 24 h at 37°C and 5% CO_2_. Afterward, the medium was replaced by 100 μL of DMEMg, containing Esc(1‐21), Esc(1‐21)ε12, and Esc(1‐21)ε20, at two different high concentrations, that is, 100 and 200 μM. The plate was incubated for 24 h, and afterward, the CCK‐8 reagent was added to each well (1:10 dilution in DMEMg), and after 90 min of incubation, the production of orange‐colored formazan dye, related to the number of living cells, was measured by evaluating the absorbance of each well at 450 nm using the microplate reader (Infinite M200; Tecan, Salzburg, Austria). The cell viability was expressed as a percentage with respect to the untreated control cells.

### 2.9 Plasma Stability Testing

Peptides were prepared as a 1 mM solution in PBS and diluted in plasma (20% v/v) that was provided by Magen David Adom Blood Donations (IRB #SMC‐13‐0954) and separated from human red blood via centrifugation to the final volume 100 μL. The mixture was incubated at 37°C for various time points. To stop further degradation, 100 μL of a stopping solution (80% ACN, 10% methanol, and 10% ddH_2_O) was added. This caused the solution to become cloudy, which was then cooled to 4°C for 1 h and centrifuged at 10,000 g for 10 min to remove plasma proteins. The supernatant (10 μL) was analyzed using an RP‐HPLC system with a Vydac C_18_ (250 × 4.6 mm, 5 μm particle size) column (Grace Discovery Sciences, Deerfield, I). The column was eluted over 30 min using a linear gradient of 20%–80% ACN in ddH_2_O containing 0.1% TFA at a flow rate of 0.6 mL/min. Absorbance was detected at 215 nm, and the decrease in the chromatographic peak area of the untreated peptide determined the percentage of the remaining peptide.

### 2.10 Large Unilamellar Vesicles Preparation

Lipid films of PEG‐PE/POPE/POPG were prepared by dissolving lipids (POPE/POPG mixture, 7:3 mol/mol + 2% PEG‐PE) in chloroform/methanol (1:1, v/v). Solvents were evaporated in a rotary vacuum system until a thin film was formed. Complete evaporation of the organic solvent was ensured by applying a rotary vacuum pump for at least 2 h. The lipid film was then hydrated with a phosphate buffer (10 mM phosphate, 140 mM NaF, 0.1 mM EDTA, pH 7.4). After 10 freeze and thaw cycles, the liposome solution was extruded 31 times by two stacked polycarbonate membranes with 50 nm pores to obtain unilamellar large vesicles (LUVs). The final lipid concentration was measured by the Stewart phospholipid assay [27].

### 2.11 Circular Dichroism Spectroscopy

Circular dichroism (CD) experiments were carried out using a Jasco J‐1500 spectrometer (Jasco International Co. Ltd., Tokyo, Japan). CD spectra were measured for each peptide at a concentration of 20 μM in buffer. LUVs were added to have a final lipid concentration of 50, 100, 200, 500, and 1000 μM. CD spectra were scanned over a range of 190–260 nm with a 1 nm data interval, scan speed 20 nm/min, bandwidth 2 nm, and averaged over 4 scans. Blank sample spectra were subtracted from the raw data, and the CD values were converted to per residue molar ellipticity([θ]) (deg cm^2^ dmol^−1^). The secondary structure content was calculated with BeStSel [28].

### 2.12 Ethics Statement

Human sample studies were carried out in strict accordance with Israeli law. All samples for experiments were conducted at the Weizmann Institute of Science and approved by Israel Institutional Review Board #SMC‐13‐0954.

## 3 Results

### 3.1 Esc(1‐21) Analogs: Synthesis and Chemical Properties

Esc(1‐21) and its analogs containing a single isopeptide bond were synthesized by an automated peptide solid‐phase synthesizer. Crude peptides were purified by RP‐HPLC, and their identity and purity were confirmed by TOF‐MS (Figure S1A) and analytical RP‐HPLC using a C_18_ column (Figure S1B), as detailed in Section 2. All peptides were 21 residues long, with a molecular weight of 2184.4 g/mol and had a net charge of +6 at neutral pH, due to the presence of the five lysine residues and an amidated C‐terminus. The primary structures and the main chemical information, including delta epsilon (Δε) from the center, retention time, and relative hydrophobicity, are reported in Table 1.

**TABLE 1:** Primary structures and chemical characteristics of Esc(1‐21) and its newly designed analogs.

Peptide	Sequence^a^	Δε from center^b^	Retention time^c^ (min)	Relative hydrophobicity^d^ (% ACN)
Esc(1‐21)	GIFSKLAGKKIKNLLISGLKG	NA	22.66	55.33
Esc(1‐21)ε5	GIFS **K** LAGKKIKNLLISGLKG	6	21.41	52.83
Esc(1‐21)ε9	GIFSKLAG **K** KIKNLLISGLKG	2	21.65	53.31
Esc(1‐21)ε10	GIFSKLAGK **K** IKNLLISGLKG	1	21.59	53.18
Esc(1‐21)ε12	GIFSKLAGKKI **K** NLLISGLKG	1	20.99	51.98
Esc(1‐21)ε20	GIFSKLAGKKIKNLLISGL **K** G	9	22.80	55.61

^a^ Underlined and bold residues are involved in isopeptide bond formation. All the peptides were amidated at their C‐terminus.

^b^ Delta epsilon (Δε) from the center is defined as the distance between the isopeptide bond position and the center of the peptide sequence.

^c^ Relative hydrophobicity is reflected by the percentage of ACN at the retention time.

^d^ The reversed‐phase HPLC retention time in the C_18_ column using a gradient of 10%–90% ACN in ddH_2_O for 40 min.

**FIGURE 1:**
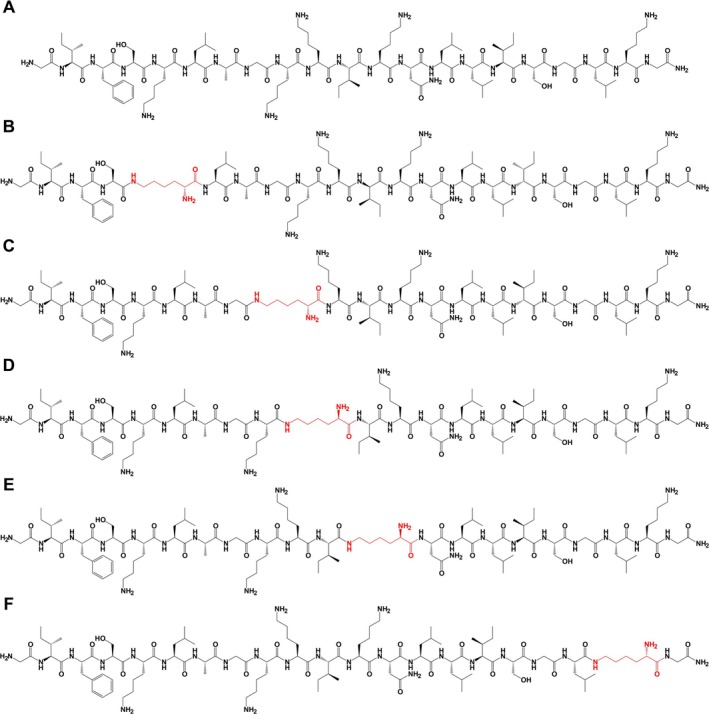
Chemical structures of Esc(1‐21) peptide and its isopeptide bond analogs. Amino acid residues in black are L‐amino acids in α‐peptide bond. Amino acid residues involved in isopeptide bond formation are highlighted in red. In the analogs, lysine residues form covalent linkages via their ε‐amino groups to the carboxyl group of the adjacent amino acid, resulting in isopeptide bonds. (A) Esc(1‐21); (B) Esc(1‐21)ε5; (C) Esc(1‐21)ε9; (D) Esc(1‐21)ε10; (E) Esc(1‐21)ε12; (F) Esc(1‐21)ε20. The chemical structures of the peptides were generated using ChemDraw software.

The chemical structure of Esc(1‐21) and the site‐specific introduction of the isopeptide bond in each designed analog are shown in Figure 1.

### 3.2 Biological Characterization

#### 3.2.1 Position‐Dependent Effects of Isopeptide Bond Incorporation on Antimicrobial Activity

The antimicrobial activity of all synthesized peptides was evaluated against a panel of Gram‐positive and Gram‐negative bacteria to determine the MIC, as reported in Table 2. The peptides were tested starting from 0.78 to 50 μM, and MIC values were compared with those already published for Esc(1‐21).

**TABLE 2:** Antimicrobial activity of Esc(1‐21) and its isopeptide bond analogs.

Strain	Antimicrobial activity (μM)^a^					
	Esc(1‐21)	Esc(1‐21)ε5	Esc(1‐21)ε9	Esc(1‐21)ε10	Esc(1‐21)ε12	Esc(1‐21)ε20
**Gram‐negative**						
*E. coli* ATCC 25922	1.56^b^	3.12	3.12	6.25	25	1.56
*P. aeruginosa* ATCC 27853	3.12^b^	12.5	6.25	12.5	25	1.56
*P. aeruginosa* ATCC 15692	2^c^	6.25	6.25	6.25	25	1.56
**Gram‐positive**						
*S. epidermidis* ATCC 12228	3.12^b^	25	12.5	25	> 50	3.12
*S. aureus* ATCC 25923	> 100^b^	> 50	> 50	> 50	> 50	> 50

^a^ Values were obtained from at least two identical readings out of three independent experiments.

^b^ Values taken from ref. [14].

^c^ Values taken from ref. [8].

As reported in Table 2, all isopeptide bond‐containing analogs were inactive against *S. aureus* , consistent with previous reports for Esc(1‐21) [14]. Meanwhile, analogs containing the isopeptide bond in positions 5, 9, and 10 showed only a slight reduction in antimicrobial activity against all the tested strains. In contrast, the antimicrobial activity of Esc(1‐21)ε12 was notably impaired by the presence of the isopeptide bond, as reflected by increased MIC values (25 or > 50 μM) against all strains. Conversely, Esc(1‐21)ε20 exhibited MIC values comparable to or slightly better than those of the parent peptide. This difference may be attributed to the position of the isopeptide bond, which, in Esc(1‐21)ε20, is closer to the C‐terminus, or possibly to changes in peptide hydrophobicity resulting from isopeptide bond formation. Specifically, Esc(1‐21)ε20 appears to have an increase in hydrophobicity that enhances its antimicrobial activity, while Esc(1‐21)ε12 likely experiences a reduction in hydrophobicity leading to lower activity (Table 1).

#### 3.2.2 Position of the Site‐Specific Isopeptide Bond Influences Membrane‐Disruptive Activity of Esc(1‐21) Analogs

**FIGURE 2:**
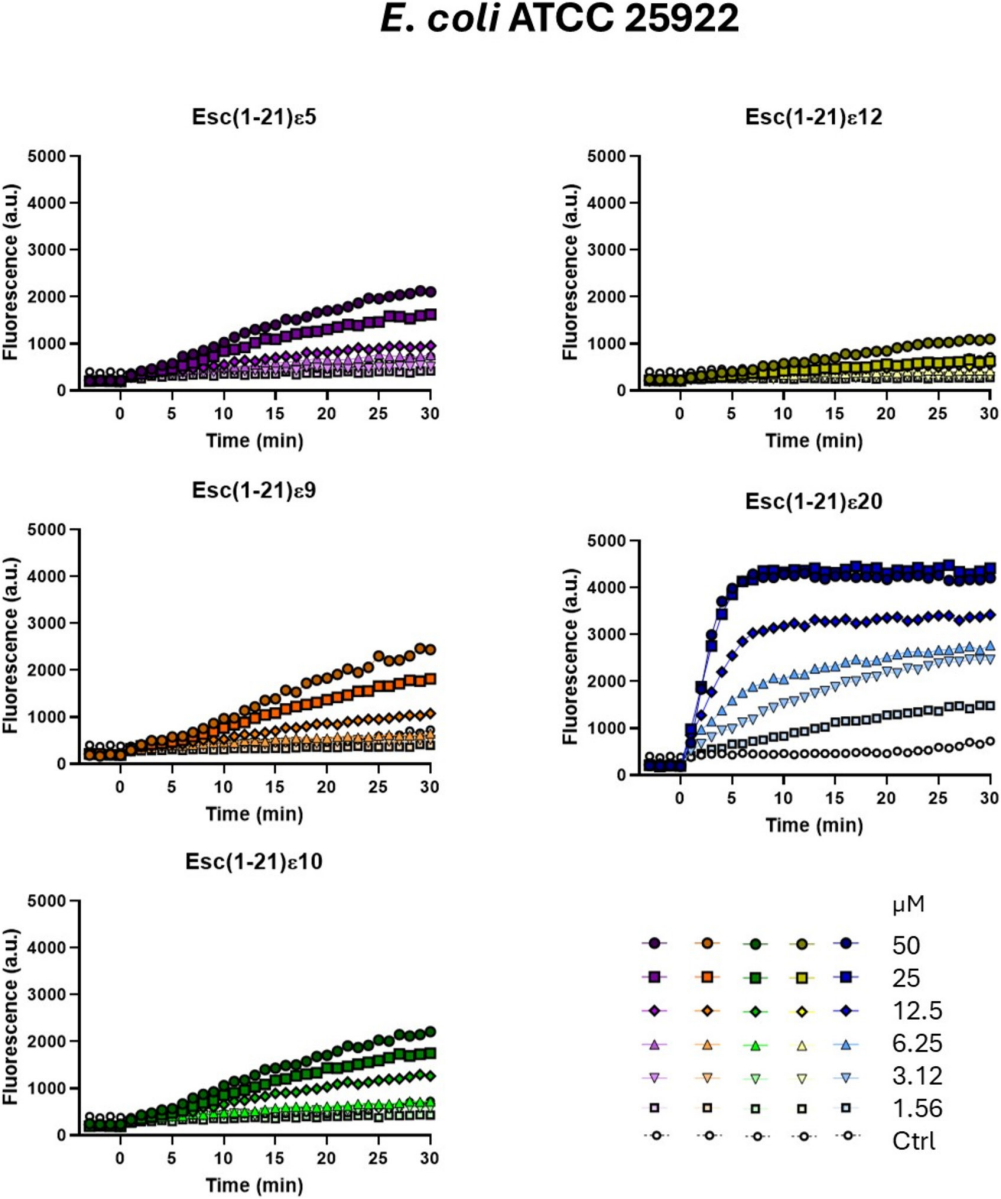
Kinetics of cytoplasmic membrane permeabilization of *E. coli* ATCC 25922 induced by the addition (time = 0) of Esc(1‐21)ε5, Esc(1‐21)ε9, Esc(1‐21)ε10, Esc(1‐21)ε12, and Esc(1‐21)ε20 at different concentrations (from 1.56 to 50 μM) evaluated by the Sytox Green assay. Controls (Ctrl) are microbial cells without the addition of any peptide. The values are from one representative experiment out of three.

**FIGURE 3:**
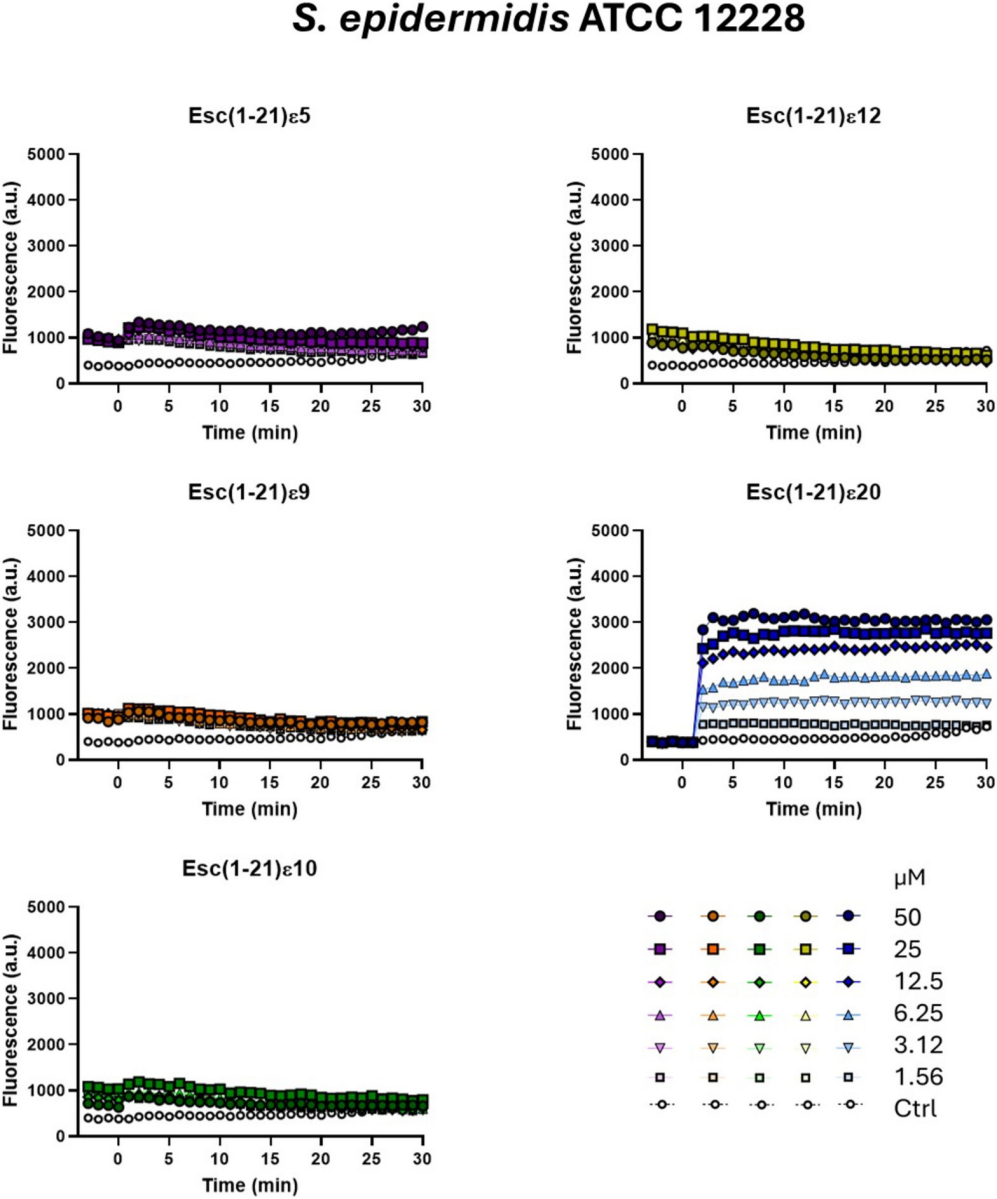
Kinetics of cytoplasmic membrane permeabilization of *S. epidermidis* ATCC 12228 induced by the addition (time = 0) of Esc(1‐21)ε5, Esc(1‐21)ε9, Esc(1‐21)ε10, Esc(1‐21)ε12, and Esc(1‐21)ε20 at different concentrations (from 1.56 to 50 μM) evaluated by the Sytox Green assay. Controls (Ctrl) are microbial cells without the addition of any peptide. The reported values are from one representative experiment out of three.

Perturbation of the cytoplasmic membrane has previously been identified as the principal mechanism underlying the antimicrobial activity of Esc(1‐21) against both Gram‐negative and Gram‐positive bacteria [14, 29]. To verify whether the weaker activity observed in Table 2 was related to a reduced capacity to perturb the cytoplasmic bacterial membranes, fluorescence studies were performed using the Sytox Green probe on two different bacterial strains, i.e. the Gram‐negative *E. coli* ATCC 25922 and the Gram‐positive *S. epidermidis* ATCC 12228. Membrane perturbation was assessed by measuring the increase in fluorescence during 30 min after peptide addition at different concentrations, ranging from 1.56 to 50 μM and results were compared with those of untreated control cells. Esc(1‐21)ε5, Esc(1‐21)ε9, and Esc(1‐21)ε10 provoked only a slight increase in fluorescence intensity against *E. coli* (Figure 2). Moreover, they did not provoke any significant increase in the fluorescence intensity after peptide addition (time = 0) against *S. epidermidis* (Figure 3). However, the most active analog, Esc(1‐21)ε20, induced a dose‐dependent membrane perturbation already from the first minutes of treatment, with kinetics comparable to those previously reported for Esc(1‐21) (Figures 2 and 3) [14, 29].

#### 3.2.3 Esc(1‐21)ε20 Exhibits Potent, Dose‐Dependent Antibiofilm Activity

**FIGURE 4:**
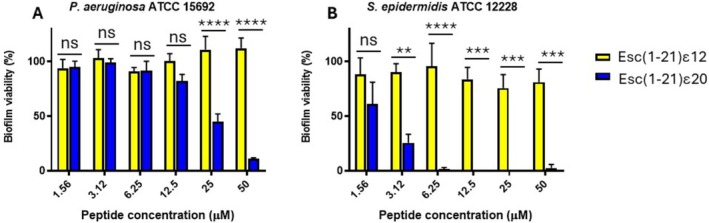
Activity of Esc(1‐21)ε12 and Esc(1‐21)ε20 against 20 h‐preformed biofilm of *P. aeruginosa* ATCC 15692 (A) and *S. epidermidis* ATCC 12228 (B) after 2 h of treatment. Biofilm viability was evaluated by measuring the reduction of the yellow MTT to its purple insoluble formazan and expressed as a percentage compared to untreated samples (bacterial biofilm not treated with the peptide, 100% viability). Data are the mean ± standard error of the mean (SEM) of three independent experiments. Statistical analysis was conducted using the two‐way ANOVA to determine the significance between the two peptides. *p < 0.05; ***p < 0.001; ****p < 0.0001; ns, not significant.

Pathogenic bacteria such as *Staphylococcus* and *Pseudomonas* pose a serious threat to human health primarily due to their ability to transition from a planktonic form (individual, free‐floating bacteria in liquid environments) to a more complex and structured form, known as biofilm [30]. In these sessile communities, bacteria are embedded in a self‐produced extracellular matrix that protects them from antibiotic molecules and facilitates surface colonization [31]. Consequently, it is crucial for antimicrobial compounds to not only inhibit or kill planktonic bacteria but also effectively eradicate biofilms [32]. Esc(1‐21) has been extensively characterized for its capability to kill both planktonic and biofilm cells [9, 14, 29], and for this reason, we evaluated the antibiofilm potency of the most active Esc(1‐21) analog i.e., Esc(1‐21)ε20 in comparison with the less active Esc(1‐21)ε12. Both peptides were tested at different concentrations against a representative Gram‐negative bacterium (i.e., *P. aeruginosa* ATCC 15692, Figure 4A) and a Gram‐positive bacterium (i.e., *S. epidermidis* ATCC 12228, Figure 4B).

Against *P. aeruginosa* , neither peptide exhibited significant activity at low concentrations (i.e., 1.56–12.55 μM). However, Esc(1‐21)ε20 showed a dose‐dependent antibiofilm activity at concentrations of 25 and 50 μM with a reduction in biofilm viability of about 60% and 80%, respectively. In contrast, Esc(1‐21)ε12 remained inactive at all concentrations against *P. aeruginosa* and was also inactive against *S. epidermidis* . In comparison, against this latter, Esc(1‐21)ε20 showed a stronger antibiofilm activity with more than 90% reduction in biofilm viability across the concentration range 6.25–50 μM.

#### 3.2.4 Site‐Specific Isopeptide Bond Switch in Esc(1‐21) Analogs Exhibit Low Hemolytic Activity

**FIGURE 5:**
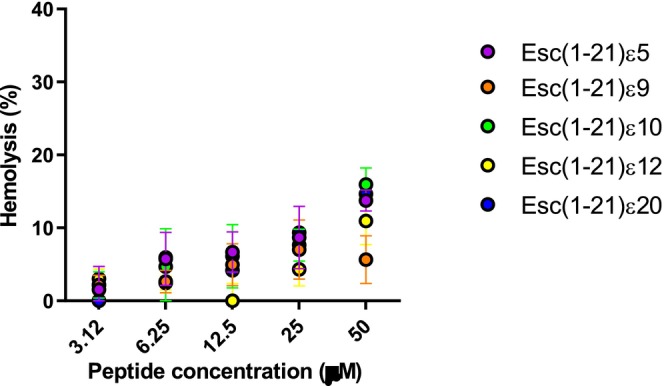
Effect of Esc(1‐21)ε5, Esc(1‐21)ε9, Esc(1‐21)ε10, Esc(1‐21)ε12, and Esc(1‐21)ε20 on mammalian RBCs after 30 min of peptide treatment at 37°C. The percentage of hemolysis was calculated with respect to the control (cells treated with vehicle). Data are the means ± SEM of three independent experiments.

To assess the potential short‐term cytotoxicity of Esc(1‐21) analogs, their ability to lyse mammalian RBCs was evaluated after 30 min of peptide treatment at 37°C. The peptides were tested at the same concentrations used in the microbiological assay (Figure 5).

All peptides showed a safe profile at all the concentrations tested, inducing less than 20% hemoglobin release even at 50 μM, a concentration that, for the most active Esc(1‐21)ε20 peptide, is 16–32 times higher than its MIC. These data are fully consistent with previous studies on Esc(1‐21) which demonstrated about 10%–15% hemolysis at the highest tested concentration of 64 μM [33].

#### 3.2.5 Esc(1‐21)ε20 Displays Reduced Cytotoxicity Compared With the Parental Peptide

**FIGURE 6:**
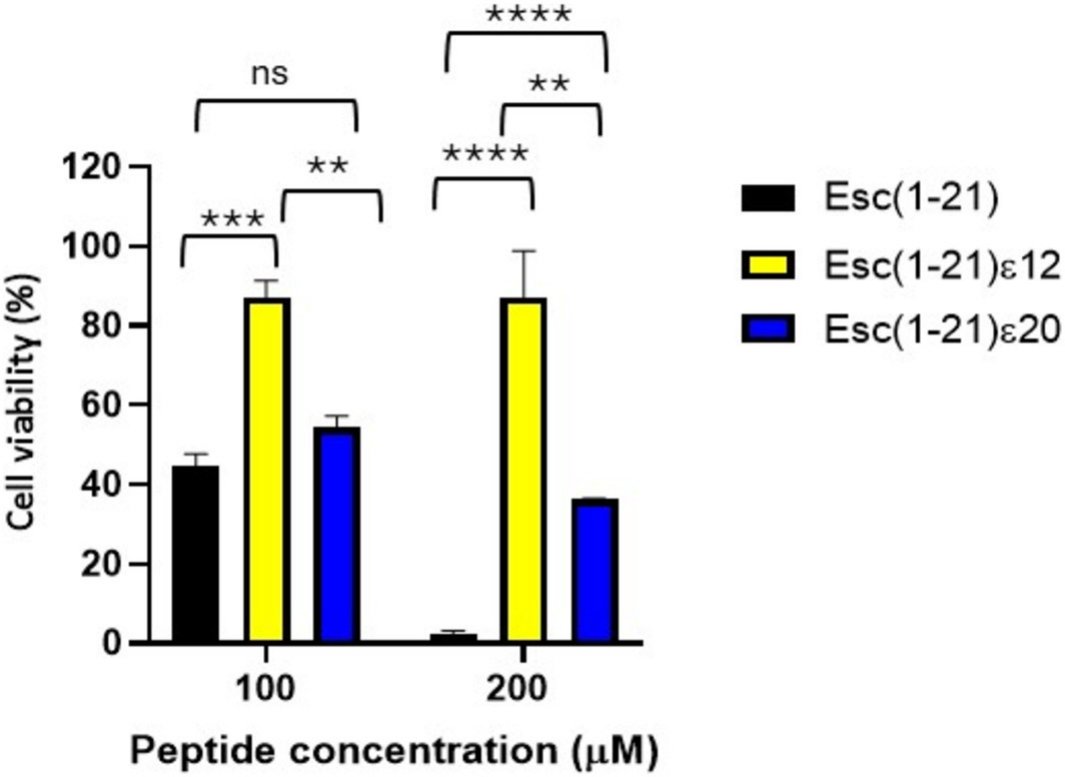
Viability of HaCaT cells after 24 h treatment with two different concentrations of Esc(1‐21), Esc(1‐21)ε12, and Esc(1‐21)ε20. Cells not treated with peptides were used as controls. All data are the means of three replicates ± SEM. Statistical analysis was conducted using two‐way ANOVA to determine the significance between the peptides. *p < 0.05; ***p < 0.001; ****p < 0.0001; ns, not significant.

The long‐term cytotoxicity of Esc(1‐21) on nucleated cells was extensively described in earlier studies, demonstrating the absence of cytotoxicity up to the concentration of 64 μM [9]. Based on these findings, the most active Esc(1‐21)ε20 analog and the less active Esc(1‐21)ε12 were tested at two higher concentrations, that is, 100 and 200 μM using the CCK‐8 assay to investigate the potential cytotoxic effect after 24 h treatment on HaCaT cells (Figure 6). The results were compared with those obtained with Esc(1‐21), considering that these two high concentrations were not tested in the previous study [9].

Esc(1‐21)ε12 peptide was not cytotoxic towards HaCaT cells at either 100 or 200 μM. In contrast, the parent peptide provoked about 50% reduction in cell viability at 100 μM and nearly complete cell death at 200 μM (Figure 6). Interestingly, Esc(1‐21)ε20 provoked a similar reduction in cell viability (~50%) at 100 μM. However, at 200 μM it exhibited lower cytotoxicity compared to the parent Esc(1‐21) with a reduction of cell viability of about 65% versus 95% of Esc(1‐21).

#### 3.2.6 Substitution With Site‐Specific Isopeptide Bond Enhances Proteolytic Stability in Human Plasma

One of the major challenges limiting the clinical application of AMPs is their susceptibility to proteolytic degradation [34]. To assess the proteolytic stability of the least and most active modified peptides, Esc(1‐21)ε12 and Esc(1‐21)ε20, as well as the parental Esc(1‐21), were incubated in fresh human plasma at 37°C for 6 and 24 h (see Section 2). The degradation profiles revealed that both isopeptide bond‐containing analogs exhibited improved stability compared to the wild‐type peptide (Table 3 and Figure S2).

**TABLE 3:** Peptide amount after 6 and 24 h incubation with human fresh plasma at 37°C.

Peptide	Peptide amount (%)^a^	
	6 h	24 h
Esc(1‐21)	30.55	15.59
Esc(1‐21)ε12	73.34	44.95
Esc(1‐21)ε20	65.61	39.64

^a^ Peptide amounts were determined by the peak areas of the RP‐HPLC relative to those of the control peptide at 0 min (set as 100%).

Notably, after 6 and 24 h, the remaining intact peptide of Esc(1‐21) was only 30.55% and 15.59%, respectively, highlighting its rapid degradation in plasma. In contrast, both Esc(1‐21)ε12 and Esc(1‐21)ε20 showed significantly higher resistance to proteolysis over the same time periods, with 73.34% and 44.95% for Esc(1‐21)ε12 and 65.61% and 39.64% for Esc(1‐21)ε20. These findings demonstrate that the site‐specific isopeptide bond switch is improving the proteolytic stability of peptides.

### 3.3 Structural Studies

#### 3.3.1 Membrane‐Induced Folding of Esc(1‐21) Analogs Depends on Isopeptide Bond Position

In principle, replacing a canonical peptide bond with an isopeptide bond could significantly affect the peptide's folding and, consequently, its ability to interact with the pathogen membrane. To investigate the impact of isopeptide bond positioning on the conformation and membrane association of the analogs, we measured the CD spectra of all the analogs in the presence of phospholipid vesicles mimicking the composition of bacterial membranes (POPE/POPG mixture, 7:3 mol/mol + 2% PEG‐PE, Figures S3–S6).

**FIGURE 7:**
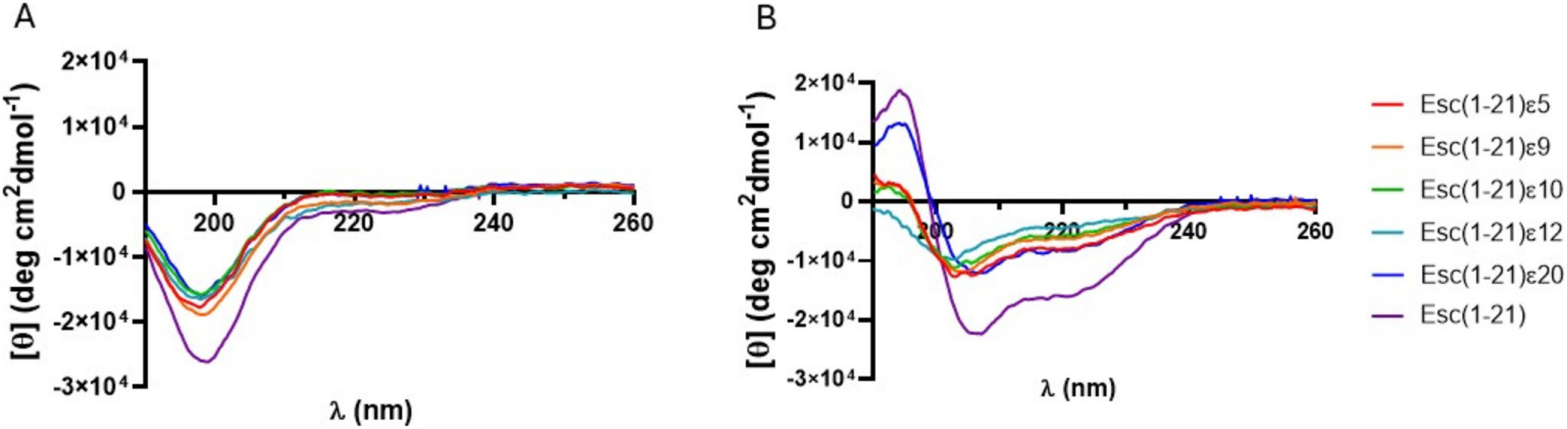
CD spectra of Esc(1–21)^2^ and its analogues (A) in aqueous solution and (B) after LUVs addition (lipid concentration 1 mM, peptide concentration 20 μM).

All the peptides are mostly unstructured in aqueous solution (Figure 7A), displaying characteristic CD spectra with a minimum in molar ellipticity near 200 nm, indicative of a random coil/other conformation. Upon addition of LUVs, only the parent peptide Esc(1‐21) and the analog Esc(1‐21)ε20 exhibited an increase in secondary structure content (Table S1).

In contrast, the remaining analogues—particularly those containing substitutions closer to the central region of the sequence (i.e., ε9, ε10, and ε12)—showed only minimal structural changes, even at the highest lipid concentration tested (i.e., 1 mM, Figure 7B), suggesting a markedly reduced capacity for membrane‐induced folding and insertion.

Unlike other analogues, Esc(1‐21)ε20 is characterized by the presence of the isopeptide bond near the C‐terminal region of the sequence. This substitution may affect the peptide folding upon membrane association only to a minor extent compared to the ε5, ε9, ε10, and ε12 analogues. These results suggest that the structural and functional integrity of membrane‐active peptides can be influenced by the precise location of isopeptide bond backbone modification.

## 4 Discussion

With the growing threat of antibiotic resistance, the discovery of new compounds with antimicrobial activity has become extremely urgent [35]. Among the candidates considered as potential new antibiotics are AMPs of innate immunity [36]. Over the years, numerous peptides have been isolated and characterized from a variety of natural sources. However, their susceptibility to proteolytic degradation results in a short half‐life [37]. In addition, although these peptides preferentially target and rapidly disrupt the anionic membranes of microorganisms, their characteristic α‐helical conformation in contact with biological membranes can lead to cytotoxicity at high concentrations [38]. It is therefore critical to explore chemical modification strategies aimed at optimizing the biological properties of these peptides. In recent years, different approaches have been employed to improve the biological properties of Esc(1‐21), a nontoxic derivative of the amphibian‐derived Esculentin‐1a [39], including the incorporation of unconventional amino acids or the application of nanotechnologies to enhance peptide stability [40].

However, the incorporation of site‐specific isopeptide bonds into the backbone sequence of nontoxic AMP, such as Esc(1‐21), has not been previously explored. This modification involves replacing conventional peptide bonds with isopeptide linkages through the ε‐amino group of lysine residues. This strategy has been used only in a few cases: in the first study, three isopeptide bonds were incorporated into two peptides, the highly toxic Amp1L and MSI‐78 (Pexiganan), generating the modified peptides Amp1EP and MSIEP, respectively. They retained key physicochemical properties while displaying reduced cytotoxicity, enhanced proteolytic stability, and minimal immune modulation. Despite adopting random coil conformations, they maintained strong antibacterial activity through membrane permeabilization [23]. In a subsequent study, a site‐specific strategy was used to introduce a single isopeptide bond into the leucine‐ and lysine‐rich Amp1L. They showed that introducing the isopeptide bond rendered the peptide nontoxic and nonhemolytic while maintaining activity and increasing protease resistance. This loss of toxicity and hemolysis is likely due to disruption of the alpha‐helical structure [22]. In another recent study, Wang et al. modified the peptide IDR‐1018 by substituting an arginine residue with lysine and introducing a lysine‐mediated isopeptide bond. The resulting peptide, 1018KI11, showed enhanced resistance to bromelain and papain digestion, along with improved antimicrobial and antibiofilm activities [20]. Taking these studies as starting points, in our work, we introduced isopeptide bonds at five distinct lysine residues (K5, K9, K10, K12, and K20). This positional switch to isopeptide bonding altered the hydrophobicity and antimicrobial activity of the resulting analogs (Tables 1 and 2). In fact, isopeptide substitutions at positions K5, K9, and K10 led to only minor reductions in antibacterial activity as indicated by the MIC values reported in Table 2. Incorporation of an isopeptide bond at the central residue K12, however, resulted in a more pronounced loss of activity, suggesting that central positioning may disrupt critical structural or functional aspects of the peptide. Conversely, modification at K20 slightly enhanced hydrophobicity and improved antimicrobial efficacy (Tables 1 and 2). Among all the analogs, Esc(1‐21)ε20 demonstrated the highest effectiveness, particularly in membrane permeabilization and antibiofilm assays (Figures 2, 3, and 4). Importantly, all isopeptide‐containing analogs exhibited low hemolytic activity up to 50 μM, indicating minimal cytotoxicity toward RBCs (Figure 5). Furthermore, susceptibility of antimicrobial peptides to degradation in human serum remains a key barrier to their therapeutic application. Given that proteases such as plasmin, kallikrein, neutrophil elastase, and cathepsin G target lysine‐rich and hydrophobic regions, we modified cleavage‐prone lysine residues to probe their role in proteolytic vulnerability [41, 42, 43, 44]. The least active Esc(1‐21)ε12 and the most active Esc(1‐21)ε20 showed enhanced proteolytic stability in human plasma compared to the unmodified Esc(1‐21), confirming that the introduction of a noncanonical peptide bond contributed to increasing peptide half‐life. The strategic incorporation of isopeptide bonds may not only disrupt canonical cleavage motifs but also offer a generalizable approach to improving peptide stability in protease‐rich physiological environments. It is well established that the conformational features are crucial for activity and cytotoxicity of cationic AMPs, and variations in the α‐helical content can influence their biological properties [45, 46, 47]. Interestingly, the relative positioning of the isopeptide bond appears to play a key role in modulating the peptide's ability to adopt an α‐helical conformation in a membrane environment. Specifically, the closer the isopeptide is to the center of the peptide sequence, the greater its disruptive effect on α‐helical folding (Figure 7). This trend is consistent with the corresponding loss of antibacterial activity and reduced cytotoxicity at high concentrations (Figure 6). Conversely, the Esc(1‐21)ε20, which retains considerable helicity due to the peripheral location of the isopeptide bond, demonstrated antimicrobial activity comparable to that of the parent Esc(1‐21), but with a lower cytotoxicity. Similar results were obtained by Wani et al., who demonstrated that peptides incorporating isopeptide bonds exhibited reduced cytotoxicity, and that introducing an isopeptide bond near the center of the sequence caused greater disruption to the AMP structure [22].

In conclusion, in this work we have introduced an isopeptide bond at the lysine residues in the backbone of the Esc(1‐21) leading to the generation of five analogues with distinct chemical and biological properties. Among these, Esc(1‐21)ε20 showed the most promising features, having (i) antimicrobial and antibiofilm activity comparable to those of the parent peptide; (ii) a lower cytotoxicity towards eukaryotic cells at high concentrations; (iii) a higher stability to proteolytic degradation. All these characteristics make Esc(1‐21)ε20 an attractive candidate for the development of new antibiotic therapies, underscoring the potential of isopeptide bond incorporation to improve AMP pharmacological properties.

## Author Contributions

B.C., D.B.H., D.R., C.V., E.K., G.C., F.C., and E.T. performed the experiments and analyzed the data. B.C. and D.B.H. wrote the manuscript. L.S., N.R.R., Y.S., and M.L.M. revised it critically.

## Conflicts of Interest

The authors declare no conflicts of interest.

## Supporting information


**Figure S1.** Mass spectrometry and analytical RP‐HPLC traces. (A) Representative MALDI‐TOF mass spectrometry spectra. (B) Analytical RP‐HPLC traces demonstrate peptide purity, with single peaks indicating high purity. RP‐HPLC was conducted using an Agilent Technologies 1260 Infinity II spectrometer with a reversed‐phase C_18_ column at a flow rate of 1.8 mL/min and monitored at 215 nm.
**Figure S2.** Isopeptide bond‐substitution enhanced the proteolytic stability of Esc(1‐21) in human plasma. HPLC chromatograms of Esc(1‐21), Esc(1‐21)ε12, and Esc(1‐21)ε20 after incubation in human plasma at 37°C for 0, 6, and 24 h. To aid visualization, traces were offset by +1, +2, and +3 min (*x*‐axis) and +100, +200, and +300 units (*y*‐axis) for 0, 6, and 24 h, respectively. Peptide separation was performed on a C_18_ column over 30 min using a linear gradient of 20%–80% ACN in ddH_2_O containing 0.1% TFA at a flow rate of 0.6 mL/min. Absorbance was monitored at 215 nm, human plasma without peptide served as a blank and the percentage of remaining peptide was quantified based on the decrease in peak area relative to the untreated sample.
**Figure S3.** CD spectra of Esc1‐21ε5 in aqueous solution (lipid concentration = 0) and after liposomes addition (lipid concentrations 50, 100, 200, 500, and 1000 μM, peptide concentration 20 μM).
**Figure S4.** CD spectra of Esc1‐21ε9 in aqueous solution (lipid concentration = 0) and after liposomes addition (lipid concentrations 50, 100, 200, 500, and 1000 μM, peptide concentration 20 μM).
**Figure S5.** CD spectra of Esc1‐21ε10 in aqueous solution (lipid concentration = 0) and after liposomes addition (lipid concentrations 50, 100, 200, 500, and 1000 μM, peptide concentration 20 μM).
**Figure S6.** CD spectra of Esc1‐21ε12 in aqueous solution (lipid concentration = 0) and after liposomes addition (lipid concentrations 50, 100, 200, 500, and 1000 μM, peptide concentration 20 μM).
**Figure S7.** CD spectra of Esc1‐21ε20 in aqueous solution (lipid concentration = 0) and after liposomes addition (lipid concentrations 50, 100, 200, 500, and 1000 μM, peptide concentration 20 μM).
**Table S1.** Estimated secondary structure of Esc(1‐21) and its analogs in aqueous and LUVs solution.

## Data Availability

The data that support the findings of this study are available from the corresponding author upon reasonable request.
