# Supplementary material for: Modulating Antimicrobial Activity and Structure of the Peptide Esc(1‐21) via Site‐Specific Isopeptide Bond Formation
Source: J Pept Sci. 2025 Aug 6;31(9):e70048. doi: 10.1002/psc.70048 (PMC12328261; doi:10.1002/psc.70048)
Supplement: Supplementary file 1 — Figure S1. Mass spectrometry and analytical RP‐HPLC traces. (A) Representative MALDI‐TOF mass spectrometry spectra. (B) Analytical RP‐HPLC traces demonstrate peptide purity, with single peaks indicating high purity. RP‐HPLC was conducted using an Agilent Technologies 1260 Infinity II spectrometer with a reversed‐phase C18 column at a flow rate of 1.8 mL/min and monitored at 215 nm. Figure S2. Isopeptide bond‐substitution enhanced the proteolytic stability of Esc(1‐21) in human plasma. HPLC chromatograms of Esc(1‐21), Esc(1‐21)ε12, and Esc(1‐21)ε20 after incubation in human plasma at 37°C for 0, 6, and 24 h. To aid visualization, traces were offset by +1, +2, and +3 min (x‐axis) and +100, +200, and +300 units (y‐axis) for 0, 6, and 24 h, respectively. Peptide separation was performed on a C18 column over 30 min using a linear gradient of 20%–80% ACN in ddH2O containing 0.1% TFA at a flow rate of 0.6 mL/min. Absorbance was monitored at 215 nm, human plasma without peptide served as a blank and the percentage of remaining peptide was quantified based on the decrease in peak area relative to the untreated sample. Figure S3. CD spectra of Esc1‐21ε5 in aqueous solution (lipid concentration = 0) and after liposomes addition (lipid concentrations 50, 100, 200, 500, and 1000 μM, peptide concentration 20 μM). Figure S4. CD spectra of Esc1‐21ε9 in aqueous solution (lipid concentration = 0) and after liposomes addition (lipid concentrations 50, 100, 200, 500, and 1000 μM, peptide concentration 20 μM). Figure S5. CD spectra of Esc1‐21ε10 in aqueous solution (lipid concentration = 0) and after liposomes addition (lipid concentrations 50, 100, 200, 500, and 1000 μM, peptide concentration 20 μM). Figure S6. CD spectra of Esc1‐21ε12 in aqueous solution (lipid concentration = 0) and after liposomes addition (lipid concentrations 50, 100, 200, 500, and 1000 μM, peptide concentration 20 μM). Figure S7. CD spectra of Esc1‐21ε20 in aqueous solution (lipid concentration = 0) and after lip [file PSC-31-e70048-s001.docx]

**Supporting information**

**Modulating Antimicrobial Activity and Structure of the Peptide Esc(1-21) via Site-Specific Isopeptide Bond Formation**

Bruno Casciaro^1,#,*^, Daniel Ben Hur^2,#^ , Daniela Roversi^3^, Carlo Vetrano^1^, Edo Kiper^2^, Giacomo Cappella^1^, Federico Carneri^3^, Eeva Tortellini^1^, Lorenzo Stella^3^, Neta Regev-Rudzki^2^, Yechiel Shai^2,*^, Maria Luisa Mangoni^1,*^.

^1^ Laboratory Affiliated to Pasteur Italia-Fondazione Cenci Bolognetti, Department of Biochemical Sciences, Sapienza University of Rome, Rome, Italy;

^2^ Faculty of Biochemistry, Department of Biomolecular Sciences, Weizmann Institute of Science, Rehovot, Israel;

^3^ Department of Chemical Science and Technologies, University of Rome Tor Vergata, 00133 Rome, Italy.

#equal contribution

Correspondence:

Bruno Casciaro, Maria Luisa Mangoni. Department of Biochemical Sciences “A. Rossi Fanelli”, Laboratory affiliated to Pasteur Italia-Fondazione Cenci Bolognetti, Sapienza University of Rome, 00185 Rome, Italy.

Email:, bruno.casciaro@uniroma1.it, [marialuisa.mangoni@uniroma1.it](mailto:marialuisa.mangoni@uniroma1.it)

Yechiel Shai. Faculty of Biochemistry, Department of Biomolecular Sciences, Weizmann Institute of Science, Rehovot 76100, Israel. Email: [Yechiel.Shai@weizmann.ac.il](mailto:Yechiel.Shai@weizmann.ac.il)


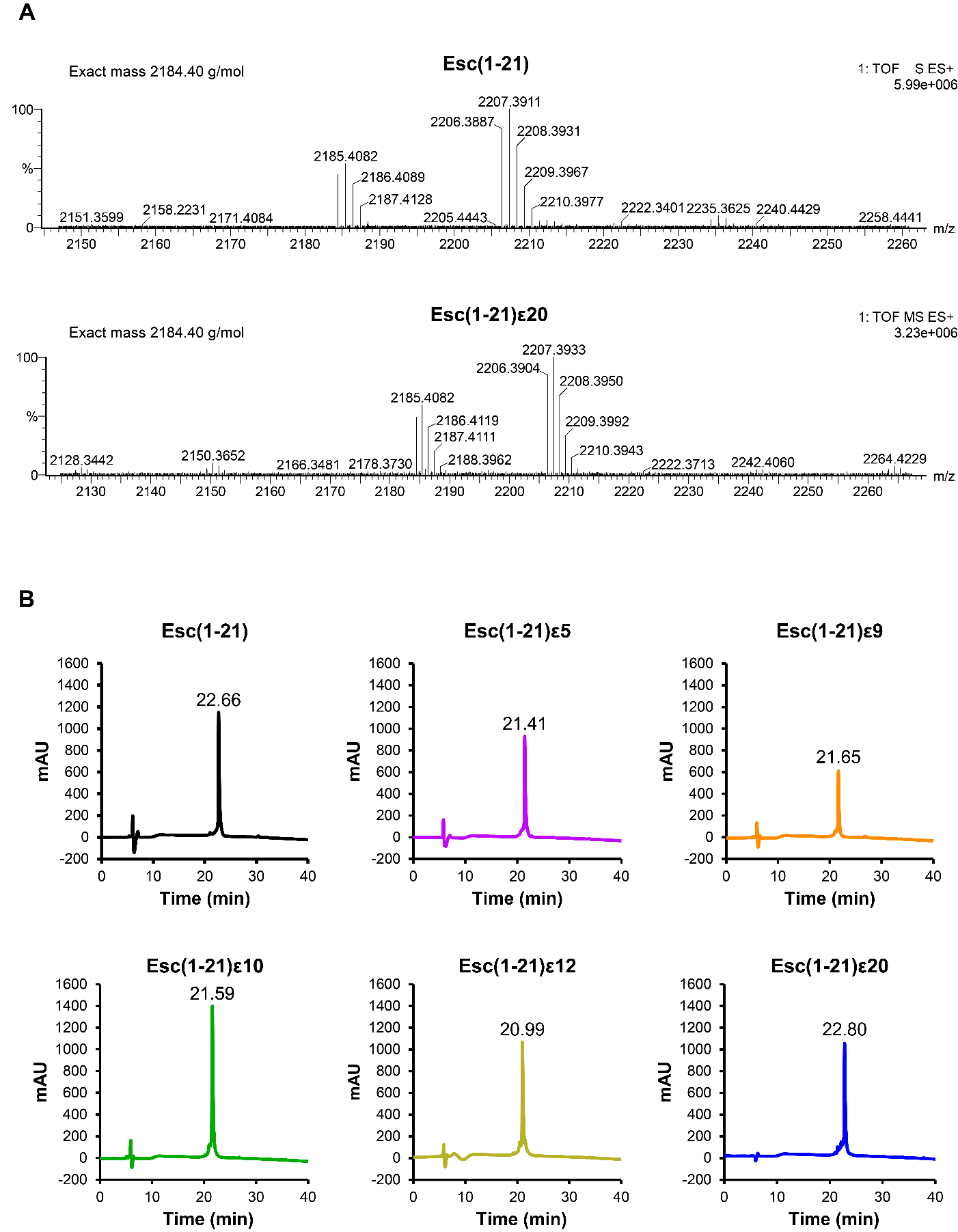


Figure S1. Mass spectrometry and analytical RP-HPLC traces. (A) Representative MALDI-TOF mass spectrometry spectra. (B) Analytical RP-HPLC traces demonstrate peptide purity, with single peaks indicating high purity. RP-HPLC was conducted using an Agilent Technologies 1260 Infinity II spectrometer with a reversed-phase C_18_ column at a flow rate of 1.8 mL/min and monitored at 215 nm.


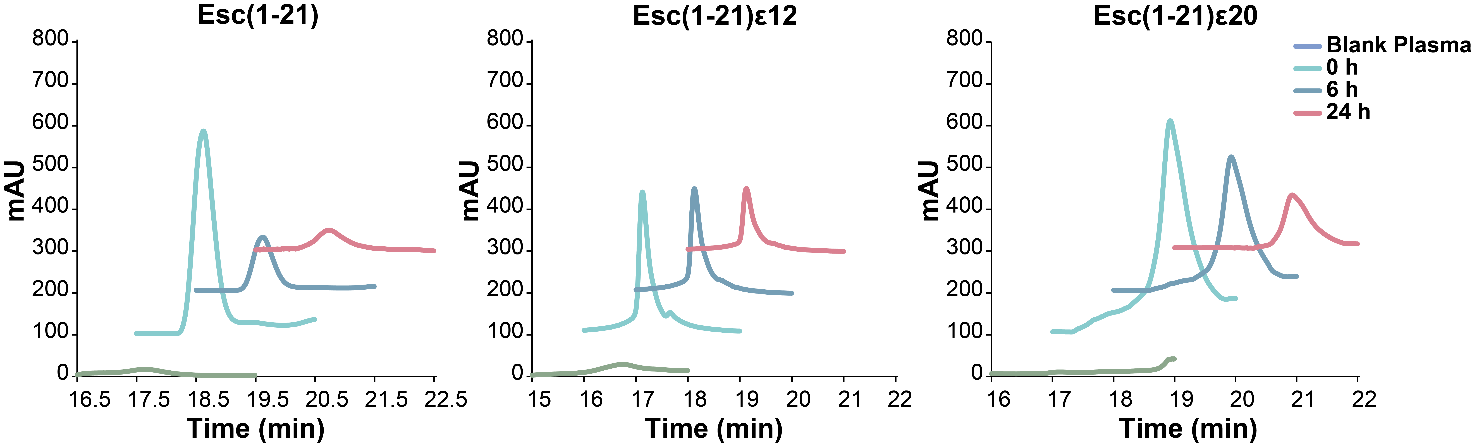


Figure S2. Isopeptide bond-substitution enhanced the proteolytic stability of Esc(1-21) in human plasma. HPLC chromatograms of Esc(1-21), Esc(1-21)ε12, and Esc(1-21)ε20 after incubation in human plasma at 37 °C for 0, 6, and 24 h. To aid visualization, traces were offset by +1, +2, and +3 min (x-axis) and +100, +200, and +300 units (y-axis) for 0, 6, and 24 h, respectively. Peptide separation was performed on a C_18_ column over 30 min using a linear gradient of 20–80% acetonitrile in ddH₂O containing 0.1% TFA at a flow rate of 0.6 mL/min. Absorbance was monitored at 215 nm, human plasma without peptide served as a blank and the percentage of remaining peptide was quantified based on the decrease in peak area relative to the untreated sample.

Figure S3. Circular dichroism spectra of Esc1-21ε5 in aqueous solution (lipid concentration =0) and after liposomes addition (lipid concentrations 50, 100, 200, 500 and 1000 µM, peptide concentration 20 μM).

Figure S4. Circular dichroism spectra of Esc1-21ε9 in aqueous solution (lipid concentration =0) and after liposomes addition (lipid concentrations 50, 100, 200, 500 and 1000 µM, peptide concentration 20 μM).

Figure S5. Circular dichroism spectra of Esc1-21ε10 in aqueous solution (lipid concentration =0) and after liposomes addition (lipid concentrations 50, 100, 200, 500 and 1000 µM, peptide concentration 20 μM).

Figure S6. Circular dichroism spectra of Esc1-21ε12 in aqueous solution (lipid concentration =0) and after liposomes addition (lipid concentrations 50, 100, 200, 500 and 1000 µM, peptide concentration 20 μM).

Figure S7. Circular dichroism spectra of Esc1-21ε20 in aqueous solution (lipid concentration =0) and after liposomes addition (lipid concentrations 50, 100, 200, 500 and 1000 µM, peptide concentration 20 μM).

Table s1. Estimated secondary structure of Esc(1-21) and its analogs in aqueous and LUVs solution

|  | [lipids] (mM) | % α-helix | % Beta | % turn | % other |
| --- | --- | --- | --- | --- | --- |
| Esc(1-21) | 0 | 0 | 33 | 20 | 47 |
|  | 1 | 34 | 8.0 | 7.0 | 51 |
| Esc(1-21)ε5 | 0 | 0 | 38 | 20 | 42 |
|  | 1 | 13 | 17 | 14 | 56 |
| Esc(1-21)ε9 | 0 | 0 | 34 | 20 | 46 |
|  | 1 | 10 | 18 | 14 | 58 |
| Esc(1-21)ε10 | 0 | 0 | 38 | 20 | 42 |
|  | 1 | 10 | 21 | 15 | 54 |
| Esc(1-21)ε12 | 0 | 0 | 33 | 18 | 49 |
|  | 1 | 6.0 | 25 | 15 | 54 |
| Esc(1-21)ε20 | 0 | 0 | 37 | 20 | 43 |
|  | 1 | 24 | 11 | 13 | 52 |
